# Structure and Phylogeny of Chloroplast and Mitochondrial Genomes of a Chlorophycean Algae *Pectinodesmus pectinatus* (Scenedesmaceae, Sphaeropleales)

**DOI:** 10.3390/life12111912

**Published:** 2022-11-17

**Authors:** Xinmei Zhao, Chenglong Liu, Lijuan He, Zhiyong Zeng, Anda Zhang, Hui Li, Zhangli Hu, Sulin Lou

**Affiliations:** 1 Shenzhen Key Laboratory of Marine Bioresource and Eco-Environmental Science, Guangdong Provincial Key Laboratory for Plant Epigenetics, Guangdong Engineering Research Center for Marine Algal Biotechnology, Longhua Innovation Institute for Biotechnology, College of Life Sciences and Oceanography, Shenzhen University, Shenzhen 518060, China; 2 Key Laboratory of Optoelectronic Devices and Systems of Ministry of Education and Guangdong Province, College of Physics and Optoelectronic Engineering, Shenzhen University, Shenzhen 518060, China; 3 Plant Introduction & Quarantine and Plant Product Key Laboratory of Xiamen City, Xiamen Overseas Chinese Subtropical Plant Introduction Garden, Xiamen 361002, China

**Keywords:** *Pectinodesmus pectinatus*, Scenedesmaceae, chlorophycean algae, organelle genome, phylogenetic analysis

## Abstract

*Pectinodesmus pectinatus* is a green alga of commercial interest in sewage purification. Clarification of its organelle genomes is helpful for genetic manipulation, taxonomic revisions and evolutionary research. Here, de novo sequencing was used to determine chloroplast genome and mitochondrial genome of *P. pectinatus* strain F34. The chloroplast genome was composed of a large single-copy (LSC) region of 99,156 bp, a small single-copy (SSC) region of 70,665 bp, and a pair of inverted repeats (IRs) with a length of 13,494 bp each separated by LSC and SSC. The chloroplast genome contained 69 protein-coding genes, 25 transfer-RNA (tRNA) genes, 3 ribosomal RNA (rRNA) genes. The mitochondrial genome was 32,195 bp in length and consisted of 46 unique genes, including 16 protein-coding genes, 27 tRNA genes and 3 rRNA genes. The predominant mutations in organelle genomes were T/A to G/C transitions. Phylogenic analysis indicated *P. pectinatus* was a sister species to *Tetradesmus obliquus* and *Hariotina* sp. within the *Pectinodesmus* genus. In analysis with CGView Comparison Tool, *P. pectinatus* organelle genomes displayed the highest sequence similarity with that of *T. obliquus*. These findings advanced research on the taxonomy and phylogeny of Chlorophyceae algae and particularly revealed the role of *P. pectinatus* in microalgae evolution.

## 1. Introduction

Phylogenetic analysis with organelle genomes leads to clarification of the evolutionary relationships [1]. Comparative organelle genomics offers a great potential perspective for species identification and provides a theoretical basis for related studies based on chloroplast and mitochondrial genomes, such as systematic evolution, genetic engineering, nuclear-cytoplasmic interaction [2,3,4,5]. With the rapid development of phylogenetic analysis and comparative genomic research, the number of organelle genomes of algae has been continuously expanding.

Photosynthetic eukaryotes convert light energy into the chemical energy in chloroplasts, which are metabolically active and semi-autonomous organelles existed in cyanobacteria, algae and plants [6]. In 1962, Ris and Plaut observed DNA filaments in chloroplasts of a chlorophyte algae classified as *Chlamydomonas* sp., uncovering the existence of genetic materials outside the nucleus of photosynthetic eukaryotes [7]. In 1978, the chloroplast genome of *Chlamydomonas reinhardtii* was successfully sequenced [8,9]. Since then, hundreds of chloroplast genome of aquatic organisms have been described [10]. The chloroplast genome differs between algal species both in term of the size and the form they take, giving an image of a diverse and quite complicated genetic system occurring inside chloroplasts [10]. Determination of chloroplast genomes of chlorophycean algae has offered crucial information for study on the origin and evolution of green algae. A famous example is a bunch of evidence from chloroplast genomes supports the hypothesis that chloroplast is an organelle descended through endosymbiosis in cyanobacteria [11,12,13]. A primary endosymbiosis with a cyanobacterium has been considered as a major evolutionary force in shaping primary plastids, which are capable of photosynthesis in both green algae and red algae. In the process of endosymbiotic evolution, the genome of the swallowed organism is greatly reduced, most of the chloroplast genes are lost or gradually transferred to the nucleus genome of the host, and the remaining genome becomes the current chloroplast genome [14]. Understanding of genetic machinery of chloroplast is one of the basic elements in order to provide the comprehensive details for studying functional activities of chloroplasts and tracking the processes inside.

Mitochondria is another two-layer membrane-coated semi-autonomous organelle, which is the place for cells to carry out aerobic respiration and produce energy. As same as chloroplasts, mitochondria have their own genetic material and genetic system. In addition to energy supplement, mitochondria are also involved in processes such as cell differentiation, information transmission, apoptosis, cell growth and cell cycle [15]. An ever-growing number of fully sequenced mitochondrial genomes have revealed the diversity in genome organization, structure, and gene content among algal species [16]. The diversity revealed by the mitochondrial genomics of green algae, along with several chloroplast genomics and nuclear genomics, will help us eventually understand the obscure phylogenetic relationships among chlorophycean algae, the cross-talk between nucleus, chloroplast, and mitochondria.

Scenedesmaceae is a family belonging to the class Chlorophyceae and includes 43 genera, such as *Hariotina*, *Pectinodesmus*, *Scenedesmus* and *Tetradesmus*. *Scenedesmus obtusus XJ-15* and *Tetradesmus obliquus* IPPAS S-2023 have showed their potential in lipid bioproduction [17]. *Coelastrella rubescens* and *Coelastrella astaxanthina* are rich in astaxanthin and canthaxanthin [18,19]. Another study found *Coelastrella sp. KGU-Y002* is another promising Scenedesmaceae algae for biosynthesis of astaxanthin, adonixanthin, and zeaxanthin [20]. In a recent evaluation of future food, Scenedesmaceae algae also displayed advantages on biomass and nutraceutical property such as polyphenols and flavonoid content [21]. Despite the ecological, economical and evolutionary importance of Scenedesmaceae [22], only a few nuclear and organelle genomes of the family Scenedesmaceae were published so far [1,23]. The genomic characterization of most species in the Scenedesmaceae family is still limited. In comparison to chloroplast genomes obtained from other algal lineages, only three complete chloroplast genome sequences of the Scenedesmaceae family are currently available in public databases.

The *Pectinodesmus pectinatus* (NCBI Taxonomy ID: 91197), formerly named as *Scenedesmus pectinatus*, is a species of chlorophycean algae in the family Scenedesmaceae and a common and important planktonic microalgae distributed globally in ponds, lakes, ditches, small puddles, various cultivation tanks and other water bodies all over the world [24,25]. *P. pectinatus* is rarely single-celled and usually composed of four to eight cells, sometimes dozens of cells [26]. *P. pectinatus* makes a high impact on environments and is economically important [27,28,29,30]. *P. pectinatu* can directly uses organic matter as a carbon and nitrogen source when it is attached to other aquatic plants to form a colloidal layer to absorb organic matter. In this way, *P. pectinatus* rapidly degrades organic matter in the water and purifies the water. More importantly, *P. pectinatus* is highly resistance to organic pollutants, and produces oxygen during photosynthesis, facilitating bacteria in the water to decompose organic matter as well. Moreover, *P. pectinatus* is easy to be cultivated in large quantities. Thus, *P. pectinatus* has great potential for use in research on water pollution control [26]. Besides, *P. pectinatus* cells can also be used as a feed for poultry since they are rich in protein and vitamins [29].

Because of differences opinion on taxonomic classification of various *Pectinodesmus* species based upon the morphological characteristics, the canonical taxonomic classification is always incomplete for species identification of the genus *Pectinodesmus*. Clarification of phylogenetic relationships is urgent for taxonomic revision and evolutionary study of *P. pectinatus*. Recently, phylogenomic studies of several strains have provided valuable insights into the phylogeny of the Chlorophyceae class, but only limited information on relationships within the Scenedesmaceae family, especially the *Pectinodesmus* genus, is available [1,23,31,32,33]. Construction of organelle genomes needs far fewer sequencing reads than constructing a draft nuclear genome. While the phylogeny studies based on -the organelle genomes are more reliable for study in evolution and specification [34]. However, the complete organelle genome information is absent for *P. pectinatus*. As for the family Scenedesmaceae, only *Tetradesmus obliquus* strain UTEX 393 chloroplast genomes (NC_008101; formerly named as *Acutodesmus obliquus* strain UTEX 393 chloroplast genome), *Tetradesmus obliquus* strain KS3-2 mitochondrial genome (NC_002254) and *Tetradesmus obliquus* strain UTEX 78 mitochondrial genomes (AF204057; formerly named as *Scenedesmus obliquus* strain UTEX 78 mitochondrial genome) are available. *Tetradesmus obliquus* strain DOE152z mitochondrial genome (CM007919), *Scenedesmus* sp. PABB004 mitochondrial genome (CM026549) and *Coelastrella* sp. MACC-549 chloroplast genome (CM031383) is assembled only but not yet annotated.

Here, to provide a better understanding of the complexity of *P. pectinatus* organelle genomes, we examined the complete sequence and genetic characterization of the chloroplast and mitochondrial genomes of *P. pectinatus* F34. Moreover, we investigated the phylogeny of *P. pectinatus* among the related genera and resolved the phylogenetic position through in-depth analysis. These results will be helpful for the organelle genetic engineering of *P. pectinatus*, which is the promising candidate for sewage purification.

## 2. Materials and Methods

### 2.1. DNA Sequencing and Genome Assembly

*Pectinodesmus pectinatus* strain F34 was obtained from the Third Institute of Oceanography (TIO) of the Ministry of Natural Resources in Xiamen, Fujian, China. *P. pectinatus* F34 was cultured in f/2 liquid medium at 22 ± 1 °C in a 12 h:12 h light-dark cycle. Total genomic DNA was extracted using a modified cetyltrimethylammonium bromide (CTAB) method and Illumina sequencing was performed by using the NEBNext UltraTM DNA Library Prep Kit for 500-bp paired-end library. Sequencing was carried out on the Illumina HiSeq2500 platform (Illumina Inc., San Diego, CA, USA). About 3.77 Gb raw data was generated with reads whose length was 250 bp. A number of potential reads were extracted from the pool of Illumina reads using BLAST searches against corresponding genomes of related species and the NOVOPlasty results [35]. The Illumina reads were obtained to perform genome de novo assembly using the Abyss package. The genome was de novo assembled with paired-end sequencing reads, which was based on the overlap and paired-end relationship. We have deposited our chloroplast genome and mitochondrial genome datasets into NCBI database and make them publicly available. The accession numbers of chloroplast and mitochondrial genomes of *P. pectinatus* F34 are NC_036668 and NC_036659, respectively.

### 2.2. Genome Annotation and Codon Usage

Preliminarily gene annotation was carried out through the online program Dual Organellar GenoMe Annotator (DOGMA) and CPGAVAS with plastid/bacterial genetic code and default conditions [36,37]. Functional annotations were performed using sequence-similarity Blast searches with a typical cut-off E-value of 10^−5^ against several protein databases: NCBI non-redundant (Nr) protein database, Swiss-Prot and Kyoto Encyclopedia of Genes and Genomes (KEGG) and Gene Ontology (GO) terms. All tRNA genes were further confirmed through online tRNAscan-SE [38,39]. The mitochondrial tRNA annotations were further confirmed by ARWEN. At the same time, using the annotation results of ARAGORN as a reference, the tRNA length was counted [40]. The graphical map was drawn with Organellar Genome DRAW (OGDRAW v1.2) [41,42]. The longest ncRNAs remained while other overlapping ncRNAs in the same area were removed through manual inspection. Manual corrections of genes for a start/stop codons and intron/exon boundaries were performed in SnapGene Viewer (from Insightful Science; available at http://snapgene.com, accessed on 16 November 2021). Codon usage, GC content, and relative synonymous codon usage (RSCU) were analyzed by CUSP program in EMBOSS (http://www.bioinformatics.nl/cgi-bin/emboss/cusp, accessed on 18 November 2021) [43]. The GC and AT asymmetry was measured in terms of GC and AT skewed distributions using the following formulae: AT-skew = (A − T)/(A + T) and GC-skew = (G − C)/(G + C). 

### 2.3. Repeat Structure and Sequence Analysis

Using online available REPuter program, the sizes and locations of forward, palindrome, reverse and complement sequences were determinedaccording to the following criteria: minimum repeat size of 30 bp, maximum computed repeats of 5000 (E-value equal to 1 × 10^−3^), and sequence identity greater than 90% (Hamming distance equal to 3) [44].The detection of simple sequence repeats (SSRs) was performed in the chloroplast genome of *P. pectinatus* F34 by using MISA software (http://pgrc.ipk-gatersleben.de/misa/, accessed on 23 November 2021). In this study, only perfect repeats were considered with basic motifs ranging from 1 to 6 nucleotides and a minimum repeat length of 10 nucleotides (for mono-), 6 nucleotides (for di-), 5 nucleotides (for tri-, tetra-, penta- and hexa-) by default. To analyse the nucleotide composition of the motif, motifs repeated on a complementary strand were considered equivalents and grouped into one motif. For instance, the motif AG is equivalent to GA, TC, CT, and so forth.

### 2.4. Comparative Genomic Analysis

The complete sequences and GenBank files of all other chloroplast genomes were downloaded from Genbank. Phylogenetic analysis was carried out based on protein-coding genes from GenBank. This study made use of 25 complete chloroplast genomes and 15 complete mitochondrial genomes of chlorophycean algae, which are listed in Table S1 Before reconstructing phylogenetic trees, both nucleotides and amino acids of protein-coding genes were subjected to concatenated alignments using MUSCLE 3.8.31 (http://www.drive5.com/muscle/, accessed on 9 December 2021) [45]. The best-fit model for nucleotides and the amino acid sequences data was selected using the Akaike Information Criterion (AIC) with jModelTest and prottest3 (http://darwin.uvigo.es/software/prottest_server.html, accessed on 10 December 2021) [46]. Phylogenetic analysis was subsequently performed using Maximum Likelihood (ML). ML analysis was conducted using the RaxML 8.1.5 software with 1000 bootstrap replicates [47]. MrBayes v3.2.2 was applied to analyze the data matrix of amino acids (BI parameters for data matrix: 1,000,000 generations, sample frequency = 100, n chains = 4, burn in value = 2500) [48]. The phylogenetic trees were drawn using the Tree View program v.1.65 and Evolview (www.evolgenius.info/evolview/, accessed on 21 December 2021) [49]. The CGView Comparison Tool (CCT) uses BLAST to compare a query genome with all other genomes and then presents the results as a circular map [50].

## 3. Results

### 3.1. Chloroplast and Mitochondrial Genomes Assembly

**Figure 1:**
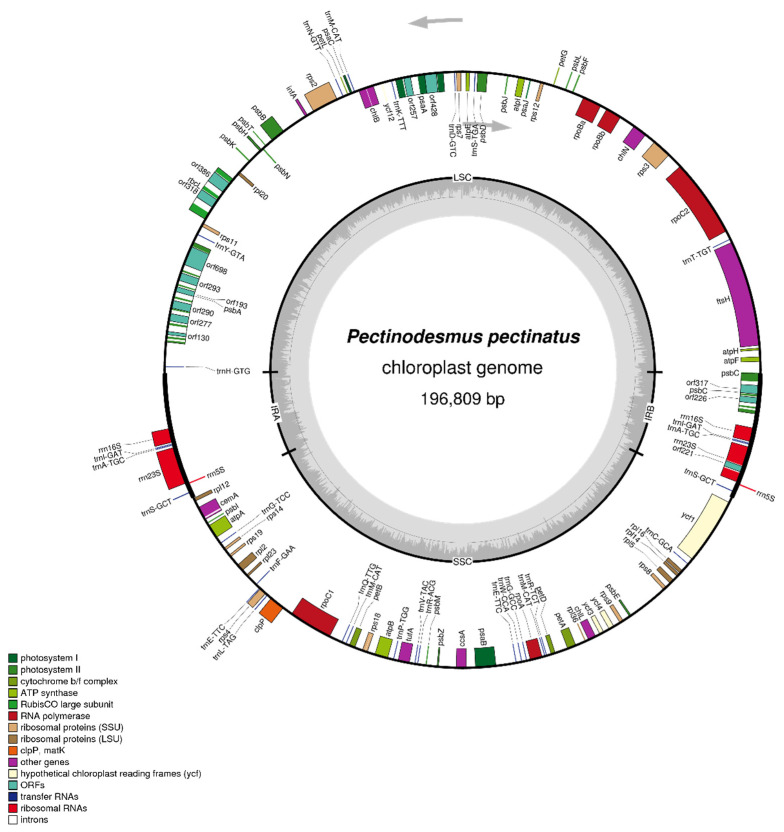
Chloroplast genome map of *P. pectinatus* F34. Genes facing inside and outside of the circle are transcribed in the clockwise and counterclockwise directions, respectively. Genes belonging to different functional groups are shown in different colors. The thick lines indicate the extent of the direct ribosomal operon repeats (IRA and IRB) that separate the genomes into large single-copy (LSC) and small single-copy (SSC) regions.

Aiming to determine the phylogenetic position of *P. pectinatus* in terms of the evolution of the chlorophycean algae and to characterize the genetic content and structure of chloroplast and mitochondrial genomes, its organelle genomes were sequenced. A total of 3.76 Gb data composed of 15,061,892 Illumina Hiseq 2500 Pair-End reads were extracted by aligning against the NOVOPlasty and Abyss assembled chloroplast genomes. Finally, reads were finally employed to assemble the *P. pectinatus* F34 chloroplast genome, whose final complete sequence with gene annotation has been deposited in the NCBI GenBank database with the accession number NC_036668. This complete chloroplast genome was finally assembled into a circular double-stranded DNA molecule of 196,809 bp in length with a typical quadripartite structure found in most terrestrial species, containing an LSC of 99,156 bp and an SSC of 70,665 bp separated by two inverted repeated rDNA sequences (IRA and IRB), each of which spanning 13,494 bp, respectively (Figure 1).

The composition of nucleotides of chloroplast genome was biased toward adenine and thymine (A + T) with a guanosine and cytosine (G + C) content of 29.94% (Table 1). The total AT content in the *P. pectinatus* chloroplast genome is 70.06% and is more than the that of Arabidopsis (63.7%), tobacco (62.2%), rice (61.1%) or maize (61.5%) (Maier et al. 1995). The GC content of LSC and SSC regions was 28.75% and 28.02%, respectively, which were lower than that of the two IR regions (39.36% and 39.36%). It can be inferred that the GC richness of the ribosomal RNA (rRNA) genes (54.88%) contribute to the high GC contents in the IR regions, which is similar to that of most green algae and land plants.

**Figure 2:**
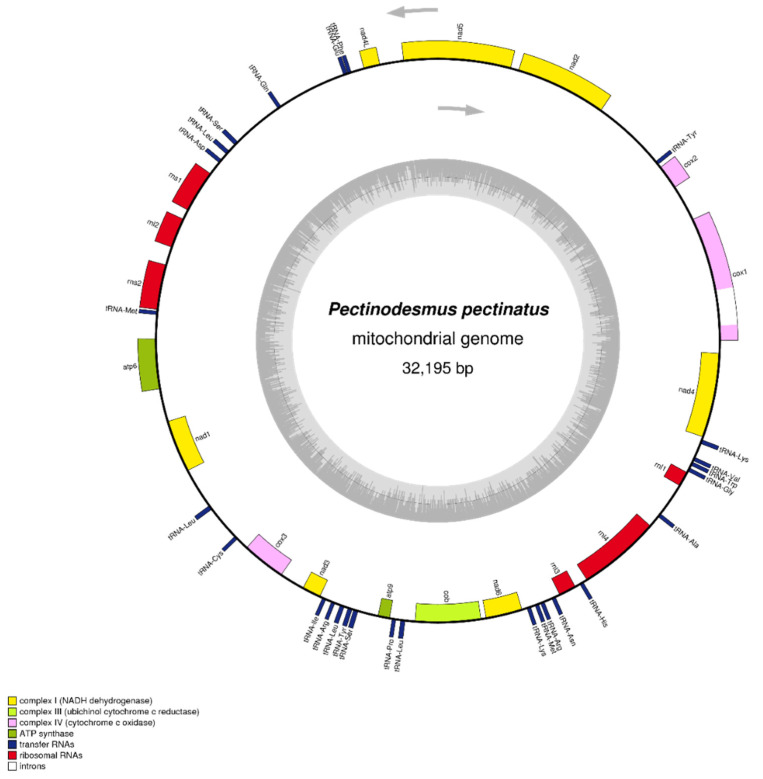
Mitochondrial genome map of *P. pectinatus* F34. Genes facing inside and outside of the circle are transcribed in the clockwise and counterclockwise directions, respectively. Genes belonging to different functional groups are shown in different colors.

In this study, Sequences of *P. pectinatus* F34 mitochondrial genome have been obtained and assembled well. As the completeness of the genome assembly is affected by conditions such as the mitochondrial DNA content and sequence complexity in the total DNA of the sample genome, for areas with complex structures or low sequencing coverage, PCR and Sanger sequencing were used for verification to ensure the completeness and accuracy of the mitochondrial genome sequence. Its final complete sequence with gene annotation has been deposited in the NCBI GenBank database with the accession number NC_036659. The complete mitochondrial genome of *P. pectinatus* was composed of 32,195 bp with a GC content of 40.78% and was more than 6 times smaller than its chloroplast genome (Figure 2). *P. pectinatus* mitochondrial genome showed more differences with regard to genome size, number of genes and introns, and repeats than the other chlorophyte algae mitochondrial genomes [51,52,53]. There appeared to be little phylogenetic conservatism of green algal mitochondrial genome sizes. The rRNA and tRNA genes of *P. pectinatus* has a total length of 4445 and 2307 bp, respectively.

### 3.2. Gene Annotation and Codon Usage

The annotation results showed that chloroplast genome of *P. pectinatus* F34 shares most gene features with other green algal chloroplast genomes. In chloroplast genome of *P. pectinatus* F34, coding regions (91,917 bp) accounted for 46.70% of the genome, and the non-coding regions, including intergenic spaces, accounted for the remaining 53.30% of the genome. In total, there were 102 genes (excluding open reading frames (ORFs)), which were scattered singly or in groups throughout the entire genome, including 69 protein-coding genes, 25 transfer-RNA (tRNA) genes and 3 ribosomal-RNA (rRNA) genes (Table 2). The gene repertoire of chloroplast genome in *P. pectinatus* is relatively homogeneous and similar to that of other green algae. A total of 69 protien-coding genes of chloroplast, including 3 ribosomal RNAs and 25 transfer RNAs showed resemblance with all other members of green algae [33,54]. Majority of genes occurred as a single copy in LSC or SSC regions, and 5 genes are duplicated in the IR regions (Table 2). Notably, three rRNA genes (rrn16S, rrn23S and rrn5S) that were duplicated in IRs, and two identical rrn16S-tRNA-Ile(ATC)-tRNA-Ala(GCA) (rrn16S-tml(GAT)-tmA(TGC)) ribosomal RNA operons were found. Interestingly, there is an insertion of orf221 between rrn23S and rrn5S in IRB region, different from the rrn23S-rrn5S cascade in IRA region. As many as 23 genes encoding tRNA molecules are located within the regions of LSC and SSC.

38 genes of *P. pectinatus* chloroplast genome were important genes involved in the photosynthesis process. Among them, there were 5 genes coding for protein subunits of the cytochrome complex, 7 genes coding for proteins being a part of the photosystem I (PS I), 16 genes coding for proteins being a part of the photosystem II (PS II), 6 genes encoding the ATPase chloroplast subunits, 12 genes encoding the NADH dehydrogenase subunits and one gene encoding the RuBisCo large subunit. In addition, 29 *P. pectinatus* chloroplast genes were related to the gene expression processes, including 5 genes encoding the chloroplast RNA polymerase subunits and 22 genes encoding protein components of the ribosomes. The transcription of chloroplast genes in angiosperm plants is carried out by two types of RNA polymerases, the bacterial-type multi-subunit RNA polymerase (PEP) encoded by the chloroplast genome and the T3/T7 phage-type RNA polymerases (NEP) encoded by the nucleus genome [55]. PEP is an enzyme composed of many subunits. In most land plants, genes coding α, β, β′ and β″ subunits of the PEP’s core are retained in the chloroplast genome, although most of the genes for PEP subunits have been transferred to the nuclear genome [56]. Similar to bacteria and most terrestrial plants, the *P. pectinatus* rpoA gene that encoding α subunit of the PEP core, together with the genes of the ribosomal proteins was organized into one operon under the control of the same promoter as well. As is known, rpoB, rpoC1 and rpoC2 genes encoded β, β′ and β″ subunits of the PEP core, respectively. Notably, there were two rpoB (rpoBa and rpoBb), two rpoC (rpoC1 and rpoC2) in chloroplast genome of *P. pectinatus*, different from some green algae and land plants in which rpoB and rpoC genes formed a separate operon designated as RPOBC [57]. In contrast, there is only one rpoB in several plant species, such as Arabidopsis, indicating the evolution factors affecting the evolution of rpoB protein. Especially, the rpoC1 is far away from rpoBa, rpoBb and rpoC2, indicating there is a rearrangement event happened in the history of evolution of *P. pectinatus* chloroplast genome.

**Figure 3:**
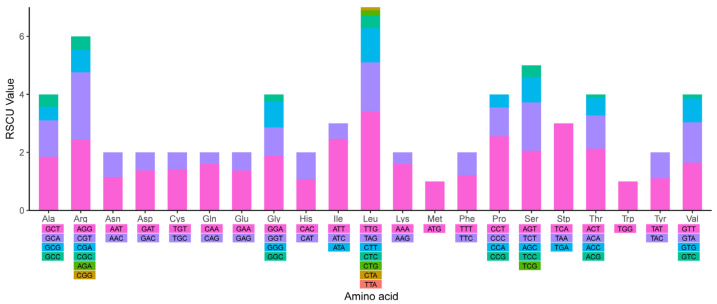
RSCU distribution map in the mitochondrial genome of *P. pectinatus* F34.

With the loss of someone within *P. pectinatus* chloroplast genome, there are only 25 tRNA genes were observed. In addition to orf221 in IRB region, another 12 ORFs were annotated, with one or two ORFs in each gene as follows: orf226 and orf317 in psbC, orf318 and orf386 in rcbL, orf257 and orf428 in psaA. A total of 6 ORFs, including orf698, orf293, orf193, orf290, orf277, orf170, were arranged in a row in a region encoding psbA. *P. pectinatus* mitochondrial genome is consist of 46 unique genes, including 13 protein-coding genes with no introns, 27 transfer-RNA (tRNA) genes and 6 ribosomalRNA (rRNA) genes (Table 3). There were no 5S ribosomal RNA genes or ribosomal genes as same as the previously reported *T. obliquus* mitochondrial genome [51]. All 13 protein-coding genes contained the same start and stop codons (i.e., ATG and TCA, respectively). There were 2 ATP synthase genes, 3 cytochrome c oxidase genes, 7 NADH dehydrogenase genes, and 1 cytochrome b gene. This was exactly consistent with the contents of *T. obliquus*. All 24 tRNA genes were encoded on the H-strand. It should be noted that 3 tRNA genes present in the *T. obliquus* were absent in *P. pectinatus*. The codon usage and codon-anticodon recognition pattern of mitochondrial genome were analyzed as well. RSCU analysis results were shown in Figure 3 The codon usage frequency distribution of mitochondrial genome is as similar as that of chloroplast genome, suggesting evolution of the gene coding mechanism within the same microalgae. Furthermore, we made use of organelle genomes of *P. pectinatus* and other species used for phylogenetic tree construction to generate heatmaps of codon usage frequency, as shown in Figure S1 (chloroplast) and Figure S2 (mitochondria). *P. pectinatus* chloroplast sequence gene annotation was annotated by CPGAVAS and DOGMA chloroplast annotation system. The annotation of the mitochondrial genome of *P. pectinatus* started with the protein and was annotated with DOGMA and ORF Finder. In annotation of the coding gene sequence, to verify the accuracy of the results and make corrections, Blastp method was used to compare with the reported coding proteins of the chloroplast and mitochondrial genomes of related species. The tRNAs with unreasonable lengths and incomplete structures were discarded, and tRNA secondary structure diagrams were generated. The rRNA annotation was validated by the Blastn method and the reported chloroplast and mitochondrial genome alignment of chlorophycean algae. All detailed gene annotation information of chloroplast genome and mitochondrial genome could be found in Table S2 and Table S3 respectively.

**Figure 4:**
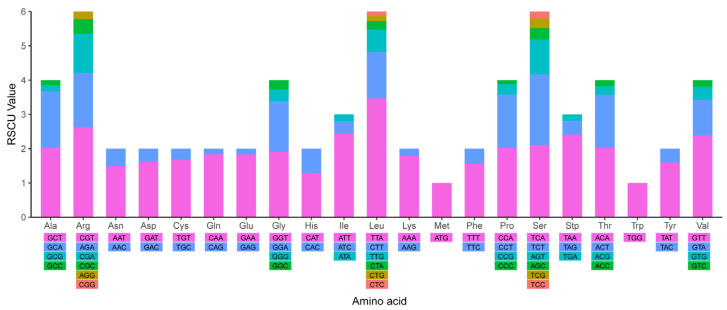
RSCU distribution map in the chloroplast genome of *P. pectinatus* F34.

Chloroplast and mitochondrial codon usage are biased (Codon Usage Bias), and codon bias will affect gene expression to a certain extent and reflect the evolutionary relationship of species. Using SCGene’s integrated Codon Usage analysis process, the codon usage and codon-anticodon recognition pattern of *P. pectinatus* chloroplast genome was analyzed. A total of 30,558 codons encoding 69 protein-coding genes were detected in the chloroplast genome. The 25 unique tRNA genes included codons for all 20 amino acids necessary for biosynthesis. The detailed codon usage frequency of chloroplast genome and mitochondrial genome were quantified by RSCU value and integrated into the distribution map as shown in Figure 3 and Figure 4, respectively. The corresponding frequency distribution maps of codon usage bias of organelle genomes of *P. pectinatus* were shown in Figures S3 and S4. The detail RSCU values of chloroplast genome and mitochondrial genome were clearly listed in Tables S5 and S6. RSCU analysis result showed that G/C contents at the first, second and third codon positions were 22.12%, 34.68% and 37.68%, respectively (Table 1). A/T was significantly enriched at the first codon position, which is common for an extremely AT-rich chloroplast genome.

### 3.3. AT-Skew and GC-Skew

In the most bacterial genomes, mitochondrial genomes, and chloroplast genomes, there are significant differences in base composition between heavy and light chains, which are called “AT (AT-skew)” and “GC (GC-skew)”. GC skew = (G − C)/(G + C), which are used to measure the relative contents of G and C. AT skew = (A − T)/(A + T), which are used to measure the relative contents of A and T. The deviation rate of GC and AT were calculated. In *P. pectinatus*, the percentage of AT reached up to 70.06% in the entire chloroplast genome (Table 4). *AT-skew and GC-skew* of the entire chloroplast genome were recorded as 0.51% and −0.69%, respectively. Interestingly, AT% of the protein-coding gene were 68.51%, but AT% (3rd) of the protein-coding gene was calculated as 62.32%.

### 3.4. SSR Identification

Microsatellites, also referred to as simple sequence repeats (SSRs), are short tandem repetitive DNA sequences that are ubiquitously present over genomes. Owing to their abundance and high-degree of polymorphism in the number of repeats, SSR research has found numerous applications such as genetic diversity, construction of genetic linkage map, variety identification and molecule marker assistant breeding [58]. In the current study, the distribution, density, repeat and motif structure of SSRs in the chloroplast genome of *P. pectinatus* were determined for use in future studies. These SSRs were nonrandomly distributed across different genomic fractions. A total of 73 perfect microsatellites were identified, with an overall density of 377.05 SSRs/Mb (Table S4). That SSRs were nonrandomly distributed across different genomic fractions.

Among these SSRs, 3 (4.11%) and 6 (8.22%) were located in the exons and introns regions, respectively, whereas 64 (87.67%) were found in the intragenic regions. Interestingly, there were much more intron-localized SSRs than exon-localized SRRs, indicating the effects of elective pressure on nucleotide. The repeatition length of the whole chloroplast genome ranged in size from 8 to 15 bp, all belonging to class II SSRs (short SSRs < 20 bp). 10 bp and 11 bp were the predominant repeat lengths in different regions. In the *P. pectinatus* chloroplast genome, the total amount of the mononucleotides (not shorter than 8 bp) was 71, which represented the highest portion (97.2%) followed by dinucleotide repeats (2.74%). All the mononucleotides were composed of A/T. These results were consistent with previous findings that SSRs in chloroplast generally consist of short polyA/T repeats. The longest poly-A and poly-T structures were a 13 A-repeat and a 13 T-repeat, respectively. Overall, the base composition of the SSR motifs showed a strong bias to AT-rich in the *P. pectinatus* chloroplast genome, consistent with other chloroplast studies. The strong A/T bias exhibited in chloroplast SSRs also contributed to the A/T enrichment in the *P. pectinatus* chloroplast genome.

### 3.5. Phylogenetic Analyses

**Figure 5:**
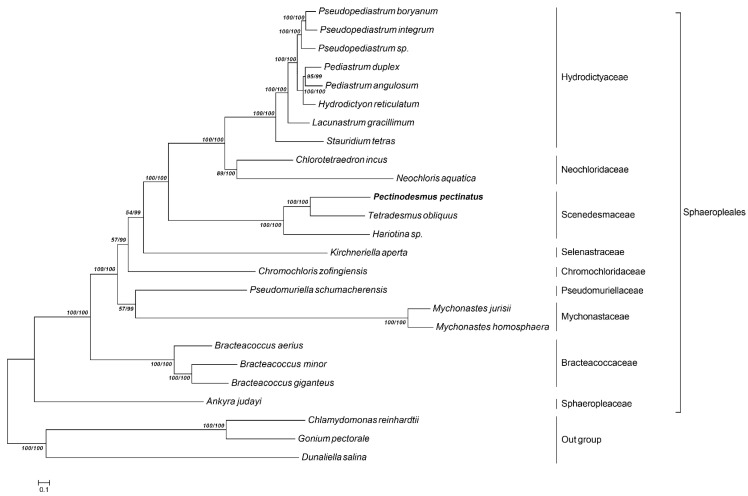
Phylogenetic tree of chloroplast nucleotide constructed by ML/BI method.

Studies on chloroplast phylogenomics of chlorophycean algae have provided valuable insights into the phylogeny of green algae, but only limited information on relationships involving the genus *Pectinodesmus* is available. Most phylogenomic studies have used one or more chloroplast genes or plastid ribosomal RNA operon to resolve the genetic relationships among green algal lineages, but a set of single-copy orthologous genes were also used. In this study, to resolve the phylogeny of *P. pectinatus* and to examine its chloroplast genome, we constructed a phylogenomic tree among chloroplast genomes of 25 representative species whose chloroplast genomes were reported previously, with commonly shared single-copy genes employing the LG + I + G model and 500 bootstrap replicates under the maximum-likelihood (ML) inference. This ML tree was largely consistent with traditional taxonomy and recent phylogenetic relationships within phylum (Figure 5). The *Coelastrella* sp. MACC-549 chloroplast genome (CM031383.1) in the NCBI database was excluded, considering that it was not yet annotated clearly.

A phylogenomic tree consisting of chloroplast genomes of 16 species was constructed. In the phylogenetic tree generated from chloroplast genome, *P. pectinatus* were falling under Sphaeropleales group. Our broadly sampled phylogenies suggested that *T. obliquus* appeared as a sister group to *P. pectinatus*, and *Hariotina* sp. was placed as a sister of *T. obliquus* and *P. pectinatus*. Together, *P. pectinatus*, *T. obliquus* and *Hariotina* sp. formed a monophyletic clade as Scenedesmaceae with almost full support, which displayed a kinship to the clade of Neochloridaceae, which is consist of *Chlorotetraedron incus* and *Neochloris aquatica*. Neochloridaceae and Selenastraceae were sister groups of Scenedesmaceae. Moreover, a clear separation between Selenastraceae and Scenedesmaceae among phylogenetic tree were observed, and the Selenastraceae are separated from Chromochloridaceae. Besides, the results showed Hydrodictyaceae is located separately from the Scenedesmaceae lineage with two distinct clusters nested inside, suggesting that Scenedesmaceae lineage independently compared with Hydrodictyaceae. It should be noted that *C. reinhardtii, Conium pectorale* and *Dunaliella salina* formed a monophyletic clade located outside as outgroup. In Sphaeropleales, *Ankyra judayi* is separated as Sphaeropleaceae that is the group most close to outgroup including Chlamydomonas, Conium and Dunaliella. Following, Bracteacoccaceae represented by *Bracteacoccus aerius*, *Bracteacoccus minor* and *Bracteacoccus giganteus* separated from Mychonastaceae, followed with Pseudomuriellaceae. Clearly, all Bracteacoccaceae, Mychonastaceae and Pseudomuriellaceae appear as some far branches to Scenedesmaceae. In conclusion, we concluded that Scenedesmaceae lineages are not monophyletic and diverged considerably deeper than the simple diversification from a single green alga.

**Figure 6:**
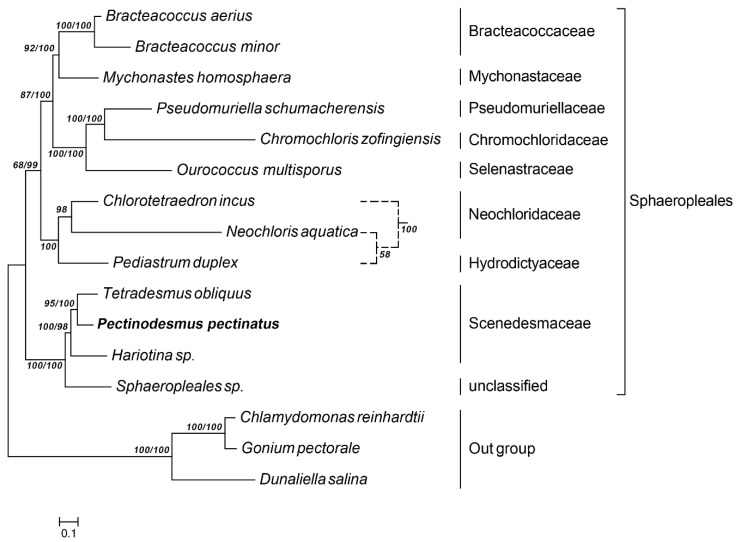
Phylogenetic tree of mitochondrial nucleotide constructed by ML/BI method; the dotted line indicates the branches built with the chloroplast amino acid.

Phylogenetic analysis of *P. pectinatus* was based on the complete mitochondrial genome data of 15 other green microalga species. In the same way, *C. reinhardtii, G. pectorale* and *D. salina* served as the outgroup. As expected, similar patterns were observed when a ML tree was constructed from alignment of mitochondrial genome sequences. In agreement with the genetic relationships observed in analysis of chloroplast genomes, *C. reinhardtii, G. pectorale* and *D. salina* constitute a monophyletic clade with outside location. Similarly, *P. pectinatus* was observed to have a closer genetic relationship with *T. obliquus* with strong bootstrap support than with any other examined species (Figure 6). According to this phylogenetic tree, *P. pectinatus*, *T. obliquus* and *Hariotina* sp. from the Scenedesmaceae showed a high degree of syntenic conservation, indicating they share a close phylogenetic affinity. Neochloridaceae is also located separately from the Scenedesmaceae lineage. *C. incus* clustered with *N. aquatica* that is another green alga from Neochloridaceae and with *Pediastrum duplex* from Hydrodictyaceae. All of these three green algae were classified into a single clade, which separated from Scenedesmaceae. Among the Sphaeropleales, a clear separation was formed between Bracteacoccaceae and Mychonastaceae, followed by the *Pseudomuriella schumacherensis* and *C. zofingiensis*, indicating Chromochloridaceae separating from Pseudomurielllaceae and Mychonastaceae. The presence of those branches and hairs are useful characteristics for distinguishing taxa.

Those phylogenomic studies recovered the phylogenomic relationship among Scenedesmaceae lineages and ordinal clades of chlorophycean algae lineages, with complete information of organelle genomes of *P. pectinatus*. There are few molecular phylogenetic studies involving the genus *Pectinodesmus*. We further used the chloroplast and mitochondrial genomes of *P. pectinatus* as a tool to unravel its evolutionary position among different Chlorophyceae groups and within Scenedesmaceae family.

### 3.6. CCT Map

Comparing the differences of organelle genomes at the genome level can reflect the biological origin and evolutionary relationship of the target species to a certain extent. According to the genetic relationship of species evolution, the chloroplast or mitochondrial genome of the species close to *P. pectinatus* was selected to construct a *CCT Map* by CGView Comparison Tool. The results are shown in Figure S3 (chloroplast) and Figure S4 (mitochondria). The sequence identity between the of *P. pectinatus* and other representatives of the chlorophycean algae was shown clearly. It appeared that both mitochondria and chloroplasts were not strongly conserved in the selected chlorophycean algae. There were still obvious differences among species in the same family, and coding genes were more conservative than non-coding genes. These results showed that *T. obliquus* achieved the highest sequence similarity, which was consistent with the results of the phylogenetic analysis. The highest similar region across all ten chloroplast genomes occurred in the IR region. Whereas LSC and SSC regions were less conservative, and many regions with identity results were lower than 90%.

## 4. Discussion

The genus *Pectinodesmus* belongs to the family Scenedesmaceae (Sphaeropleales, Chlorophyceae). It recently formed a new genus separate from *Scenedesmus* based on the internal transcribed spacer, two consensus sequences, which were distinguished from other members of the family Scenedesmaceae [59]. There are five species in this new genus with four of them having been removed from the genus *Scenedesmus*, including *P. pectinatus* [24]. *P. pectinatus* is a typical cenobium with four or eight cells arranged linearly or twisted up to 90° to each other [26]. Information for species identification is limited about *P. pectinatus* other than its basic morphological characteristics and preliminary taxonomic status. The green algae show extensive variation in chloroplast genome size [60]. In this study, the genome structure of *P. pectinatus* is in agreement with published chloroplast genomes of land plants and green algae, in which identical long IRs containing the ribosomal operons, suggesting it was evolved from the ancestor of chlorophycean algae [1,33,61].

Consistent with previous findings in several green algal plastids, introns were completely present in protein-coding genes and tRNAs of chloroplast genome of *P. pectinatus*. It indicates that those genes suffered from election by certain factors. As summarized in Table 5, the psbC, psaA, chlB, rbcL and psbA genes were five split genes in LSC regions in chloroplast genomes. Four genes (chlB, cemA, rps18 and rrn23S) have a single intron and two genes (psaA and rbcL) have two introns (Table 5). The rps18 gene has the shortest intron, that is 23 bp only, while the psbA gene has as many as seven introns whose total length is 9966 bp. Interestingly, psbA is consist of eight exons and seven introns, possessing most introns among *P. pectinatus* chloroplast genome. It raises a challenge question why *P. pectinatus* psbA keeps so many introns in the long evolution history. Another two split genes, cemA and rps18, were residents in the SSC region. In addition, one of the three rRNA genes in *P. pectinatus* chloroplast genome, rrn23S is the only one containing intron in the IRs area. Because the intron was present in both duplicates, it is clear that the rrn23S got copied in chloroplast genome after the intron-exon splicing site evolved.

In comparison with other core Chlorophyta (clade comprising the Ulvophyceae, Trebouxiophyceae, Chlorophyceae, Pedinophyceae, and Chlorodendrophyceae), *P. pectinatus* have one gene encoding for components of the cell division protein (ftsH) found in other green algae (*Trebouxiophytes*, and *Pedinomonas*) but lost in some Chlorodendrophyceae chloroplast genomes [62,63]. The chloroplast envelope membrane protein (cemA) gene was conserved in the *P. pectinatus* as well. Experiments on *C. reinhardtii* knockout mutant have shown that cemA is not essential for photosynthesis or the viability of the cell but its absence increases the light sensitivity [64]. Notably, 6 ycf genes that were found in chloroplast genome of *P. pectinatus* remain elusive. Among which, 3 ycf genes could be functionally annotated with high similarity, including ycf1 (component of TIC complex), ycf3 and ycf4 (subunits of photosystem I). Apart from these ycf genes, homologous gene of ycf12 is reported as a subunit of photosystem II reaction center and is required for optimal growth in high light in *C. reinhardtii* [65].

Molecular phylogenetics are the cornerstone of current biodiversity and evolutionary research. Undoubtedly, many features conserved among mitochondrial genomes were observed, but the chloroplast genome organization is more conserved and valuable for understanding the evolutionary pattern and phylogeny of green algae. Besides, to unravel phylogenetic relationships, complete chloroplast genome sequences are a better choice than concentrating only on protein-coding sequences or conserved regions. Here our main focus was on the position of *P. pectinatus* within Chlorophyceae algae, and we have observed close phylogenetic relationships between *P. pectinatus*, *T. obliquus* and *Hariotina* sp. It was not surprising that similar patterns of clustering were obtained in the ML tree constructed from mitochondrial genome sequences as the one observed from analysis based on chloroplast genomes. However, in this study, the mitochondrial genome ML tree supported the significant separation between *P. pectinatus* and Selenastraceae. It is different from the sister relationships of chloroplast genomes of Selenastraceae and Scenedesmaceae, clear separated groups were formed for Selenastraceae represented by *Ourococcus multisporus* and Scenedesmaceae including *P. pectinatus*, *T. obliquus* and *Hariotina* sp. Results clearly showed that *P. pectinatus* and *T. obliquus* formed a separate clade within Scenedesmaceae, whereas *Hariotina* sp. was relatively distant from the other two green algae. Thus, collinearity analysis of mitochondrial genomes was performed on *P. pectinatus* and other three chlorophycean algae. The results attested that the gene rearrangements between different algal species are universal and showed that the rearrangement of *P. pectinatus* mitochondrial genome compared with other green algae (Figure S5). Interestingly but not surprisingly, the order of the mitochondrial gene in *P. pectinatus* is largely different from that of *T. obliquus*, which is the closest sister in phylogenic tree. In both evolutionary analyses, *C. reinhardtii, Gonium pectorale* and *Dunaliella salina* were classified as outgroup and they served the purpose effectively. Although the relationships of Scenedesmaceae with other major clades from Chlorophyceae remains unclear, our results now showed the genetic position of Scenedesmaceae in all green algae. Our results on *P. pectinatus* are an add-on in the understanding of the phylogenetic relationship among different Chlorophyceae species.

## 5. Conclusions

During the endosymbiosis event happened over 1.2 billion years ago, the free-living cyanobacterium started to coexist inside a eukaryotic cell, which turned out to be essential for life. Understanding of chloroplast and mitochondrial genetic machineries is key to a full explanation of the basic process in green algae. With the strong believes that these organelle genomes can play a great tool for taxonomic and phylogenetic analysis, we successfully assembled and analyzed chloroplast and mitochondrial genomes of *P. pectinatus* F34 in this study. Phylogenetic analysis based on these organelle genomes showed that *P. pectinatus* was closely related to *T. obliquus* and *Hariotina* sp. and also supported the well-known classification of different Chlorophyceae groups and families. Yet it is unclear whether current data in our hands are sufficient to finally obtain a robust tree topology for all the genus of Chlorophyceae, further study is still needed. This phylogeny would require further investigation by a broader sampling of chlorophycean algae. All in all, the sequenced complete chloroplast genome and mitochondrial genome of *P. pectinatus* F34 provides detailed fundamental molecular data for species identification, Chlorophyceae evolution and genetic manipulation, serving its potential in water-sewage industry.

## Figures and Tables

**Table 1:** Base composition in the LSC, SSC, IRA, IRB of chloroplast genome of *P. pectinatus* F34. ^1^ A(U) (%): the proportion of adenine bases; ^2^ T (%): the proportion of thymine bases; ^3^ G (%): the proportion of guanine bases; ^4^ C (%): the proportion of cytosine bases; ^5^ GC (%): the proportion of guanine bases and cytosine bases; ^6^ Length (bp): sequence length. Total: the total number of bases in the chloroplast genome; CDS: protein coding region; 1st position: the first base of the triplet codon; 2nd position: the second base of the triplet codon; 3rd position: The third base of the triplet codon.

		A(U) (%) ^1^	T (%) ^2^	C (%) ^3^	G (%) ^4^	GC (%) ^5^	Length (bp) ^6^
**LSC**		36.08	35.17	14.4	14.36	28.75	99,156
**IRA**		31.29	29.35	17.58	21.78	39.36	13,494
**SSC**		35.85	36.14	14.27	13.75	28.02	70,665
**IRB**		29.35	31.29	21.78	17.58	39.36	13,494
**Total**		35.21	34.85	15.08	14.87	34.00	196,809
**CDS**		33.84	34.67	15.83	15.66	31.49	91,917
	**1st** **position**	40.89	36.99	10.94	11.18	22.12	30,638
	**2nd** **position**	31.31	34.01	14.89	19.79	34.68	30,638
	**3rd** **position**	29.31	33.01	21.67	16.01	37.68	30,638

**Table 2:** Annotation of chloroplast functional genes of *P. pectinatus* F34. *: Duplicate gene (indicating that there are at least two duplications in the chloroplast genome).

Category of Genes	Group of Gene	Name of Gene
Self-replication	Small subunit of ribosome	rps2 rps3 rps4 rps7 rps8 rps9 rps11 rps12 rps14 rps18 rps19
	Large subunit of ribosome	rpl2 rpl12 rpl14 rpl5 rpl16 rpl20 rpl23 rpl36
	DNA-dependent RNA polymerase	rpoA rpoBa rpoBb rpoC1 rpoC2
	Ribosomal RNA genes	rrn5S * rrn16S * rrn23S *
	Transfer RNA genes	tRNA-Ala(GCA) * tRNA-Arg(CGT) tRNA-Arg(AGA) tRNA-Asn(AAC) tRNA-Asp(GAC) tRNA-Cys(TGC) tRNA-Gln(CAA) tRNA-Glu(GAA) * tRNA-Gly(GGA) tRNA-Gly(GGC) tRNA-His(CAC) tRNA-Ile(ATC) * tRNA-Leu(CTA) tRNA-Lys(AAA) tRNA-Met1(ATG) * tRNA-Met2(ATG) tRNA-Phe(TTC) tRNA-Pro(CCA) tRNA-Ser1(AGC) tRNA-Ser2(AGC) tRNA-Ser3(TCA) tRNA-Thr(ACA) tRNA-Trp(TGG) tRNA-Tyr(TAC) tRNA-Val(GTA)
Genes for photosynthesis	Photochlorophyllide reductase	chlB chlL chlN
	Large subunit of Rubisco	rbcL
	Subunits of photosystem II	psbA psbB psbC psbD psbE psbF psbH psbI psbJ psbK psbL psbM psbN psbT psbZ ycf12
	Subunits of photosystem I	psaA psaB psaC1 psaC2 psaJ ycf3 ycf4
	Subunits of ATP synthase	atpA atpB atpE atpF atpH atpI
	Subunits of cytochrome	petA petB petD petG petL
Other genes	Elongation factor Tu	tufA
	Envelope membrane protein	cemA
	C-type cytochrome synthesis gene	ccsA
	Cell division protein	ftsH
	Protease	clpP
	Translation initiation factor	infA
	Component of TIC complex	ycf1

**Table 3:** Annotation of mitochondrial functional genes of *P. pectinatus* F34.

Category of Genes	Group of Gene	Name of Gene
Self-replication	Small subunit of ribosome	rns1 rns2
	Large subunit of ribosome	rnl1 rnl2 rnl3 rnl4
	Transfer RNA genes	tRNA-Ala tRNA-Arg1 tRNA-Arg2 tRNA-Asn tRNA-Asp tRNA-Cys tRNA-Gln tRNA-Glu tRNA-Gly tRNA-His tRNA-Ile tRNA-Leu1 tRNA-Leu2 tRNA-Leu3 tRNA-Leu4 tRNA-Lys1 tRNA-Lys2 tRNA-Met1 tRNA-Met2 tRNA-Phe tRNA-Pro tRNA-Ser1 tRNA-Ser2 tRNA-Trp tRNA-Tyr1 tRNA-Tyr2 tRNA-Val
Other genes	cytochrome c oxidase	cox1 cox2 cox3
	NADH dehydrogenase	nad1 nad2 nad3 nad4 nad5 nad6 nad4L
	cytochrome b	cytb
	Subunits of ATP synthase	atp6 atp9

**Table 4:** Base composition in the organelle genomes of *P. pectinatus* F34. AT%: Represents the AT content of the entire genome; GC-skew: GC offset; the calculation formula is GC-skew = (G − C)/(G + C); AT-skew: Represents AT offset; the calculation formula is AT-skew = (A − T)/(A + T); Length (aa): indicates the number of amino acids encoded by all exons; AT% (all): indicates the AT content of all coding regions; AT% (3rd): After all coding regions are connected together, the AT content of a new sequence composed of the third base every 2 bases is taken; Length (bp): indicates the number of bases that all tRNAs are connected together; AT% (t): indicates the AT content of the sequence after all tRNA fragments are joined together.

	Entire Genome				Protein-Coding Gene			tRNAs	
	Length	AT%	GC-Skew	AT-Skew	Length (aa)	AT% (all)	AT% (3rd)	Length (bp)	AT% (t)
**Chloroplast**	196,809	70.06	-0.69	0.51	30,558	68.51	62.32	2184	46.57
**Mitochondrial**	32,195	59.22	0.0095	0.0036	4232	56.6	56.63	1810	51.55

**Table 5:** Split genes in chloroplast genome of *P. pectinatus* F34. Location: represents the area where the gene is located; Exon I, II, III: respectively represent the first, second, and third exons of the gene; Intron I and Intron II, respectively represent the first and second introns of the gene. * Duplicate gene (indicating that there are at least two duplications in the chloroplast genome).

Gene	Location	Exon I (bp)	Intron I (bp)	Exon II (bp)	Intron II (bp)	Exon III (bp)	Intron III (bp)	Exon IV (bp)	Intron IV (bp)	Exon V (bp)	Intron V (bp)	Exon VI (bp)	Intron VI (bp)	Exon VII (bp)	Intron VII (bp)	Exon VIIII (bp)
psbC	LSC	882	1497	74	1223	72	293	337								
psaA	LSC	707	1742	628	1416	654										
chlB	LSC	799	48	800												
rbcL	LSC	460	1921	240	1790	731										
psbA	LSC	174	305	105	1324	116	1520	28	1213	91	1304	47	1479	73	2821	431
cemA	SSC	90	262	1158												
rps18	SSC	250	27	317												
rrn23S *	IRs	2051	938	967												

## Data Availability

We deposited our cp genome and mt genome datasets into NCBI database and make them publicly available. The accession numbers of chloroplast and mitochondrial genomes of *P. pectinatus F34* are NC_036668 and NC_036659, respectively.

## Supplementary Materials

The following are available online at https://www.mdpi.com/article/10.3390/life12111912/s1, Figure S1: Heat map of codon usage frequency of cp genome; Figure S2: Heat map of codon usage frequency of mt genome; Figure S3: CCT map of cp genomes; Figure S4: CCT map of mt genomes; Figure S5: Rearrangement of mitochondrial genomes of *P. pectinatus* in comparison to other 3 chlorophycean algae; Table S1: Information of Organelle Genomes; Table S2: Gene annotation information of chloroplast genome; Table S3: Gene annotation information of mitochondrial genome Table S4: Identification of SSR.
