# Supplementary material for: Structure and Phylogeny of Chloroplast and Mitochondrial Genomes of a Chlorophycean Algae Pectinodesmus pectinatus (Scenedesmaceae, Sphaeropleales)
Source: Life (Basel). 2022 Nov 17;12(11):1912. doi: 10.3390/life12111912 (PMC9698225; doi:10.3390/life12111912)
Supplement: Supplementary file 1 [file life-12-01912-s001.zip › Figure S5.pdf]

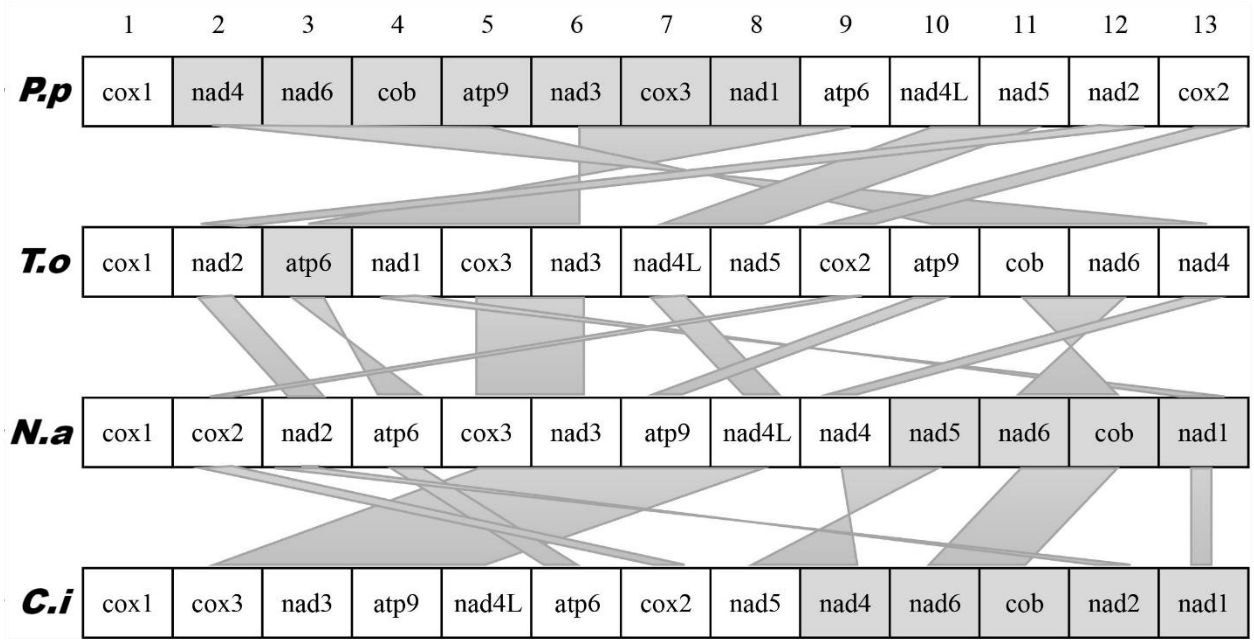

Figure S5. Rearrangement of mitochondrial genomes of *P. pectinatus* in comparison to other 3 chlorophycean algae.

The genes marked in white are the genes on the heavy chain (outer circle in the map), and the genes marked in gray are the light chain (genes in the inner circle in the map). The figure only reflects the relative position of genes, not the length and the similarity between species. *P.p*: *Pectinodesmus pectinatus*; *T.o*: *Tetradesmus obliquus*; *N.a*: *Neochloris aquatica*;*C.i*: *Chlorotetraedron incus*.
