# Supplementary figures and images for: Structure and Phylogeny of Chloroplast and Mitochondrial Genomes of a Chlorophycean Algae Pectinodesmus pectinatus (Scenedesmaceae, Sphaeropleales)
Source: Life (Basel). 2022 Nov 17;12(11):1912. doi: 10.3390/life12111912 (PMC9698225; doi:10.3390/life12111912)

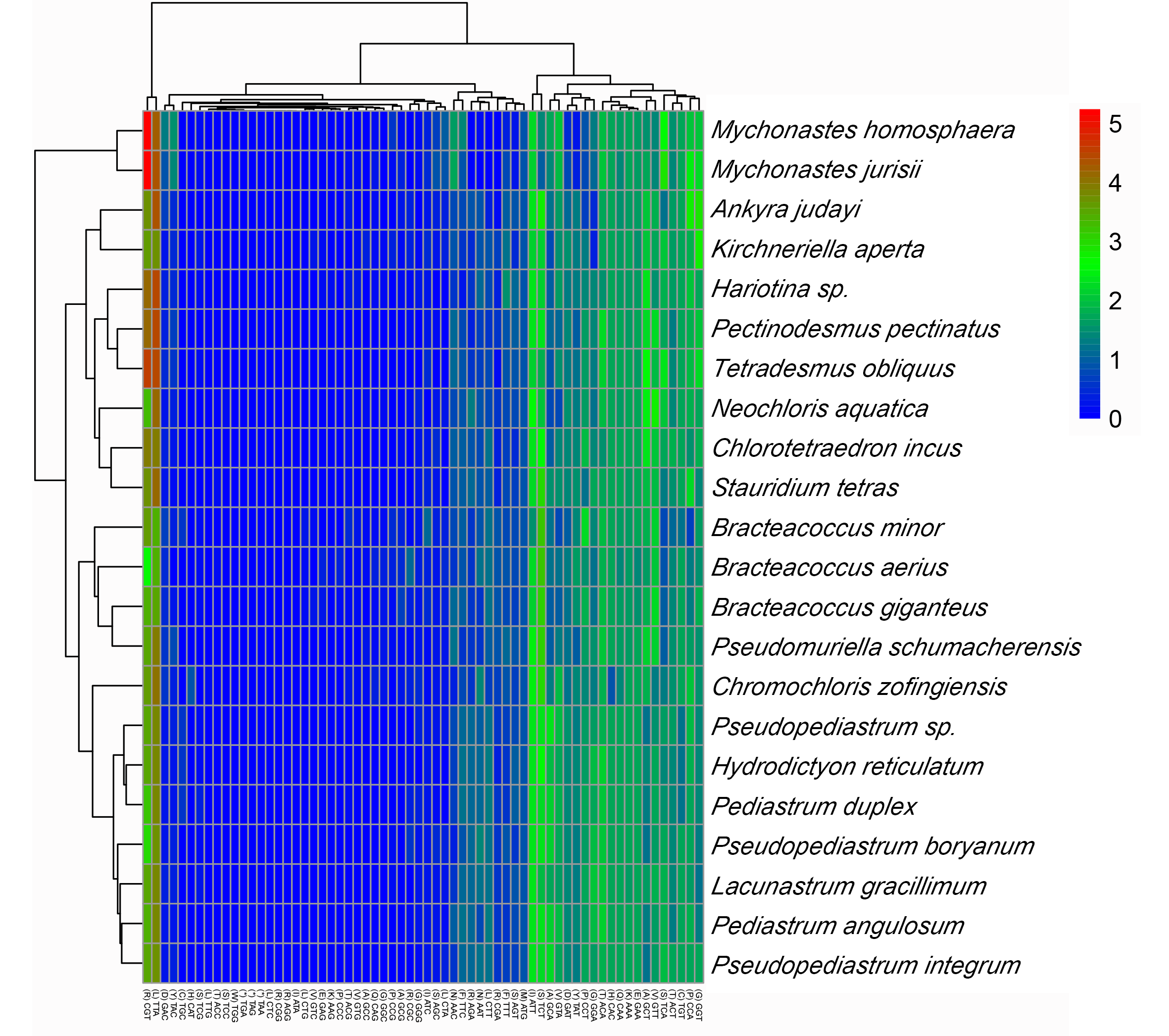

Supplement: Supplementary file 1 [file life-12-01912-s001.zip › Figure S1.tif]

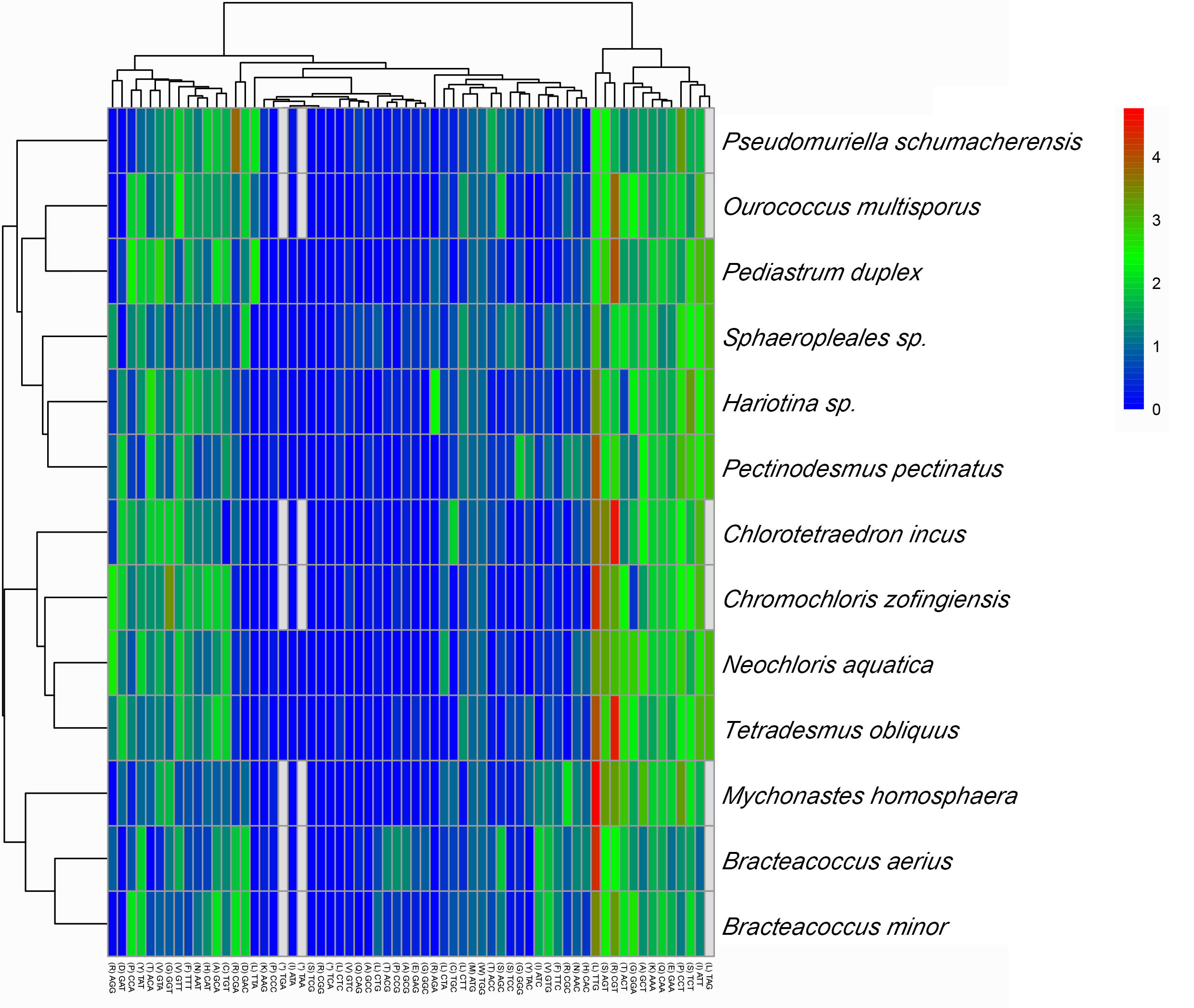

Supplement: Supplementary file 1 [file life-12-01912-s001.zip › Figure S2.tif]

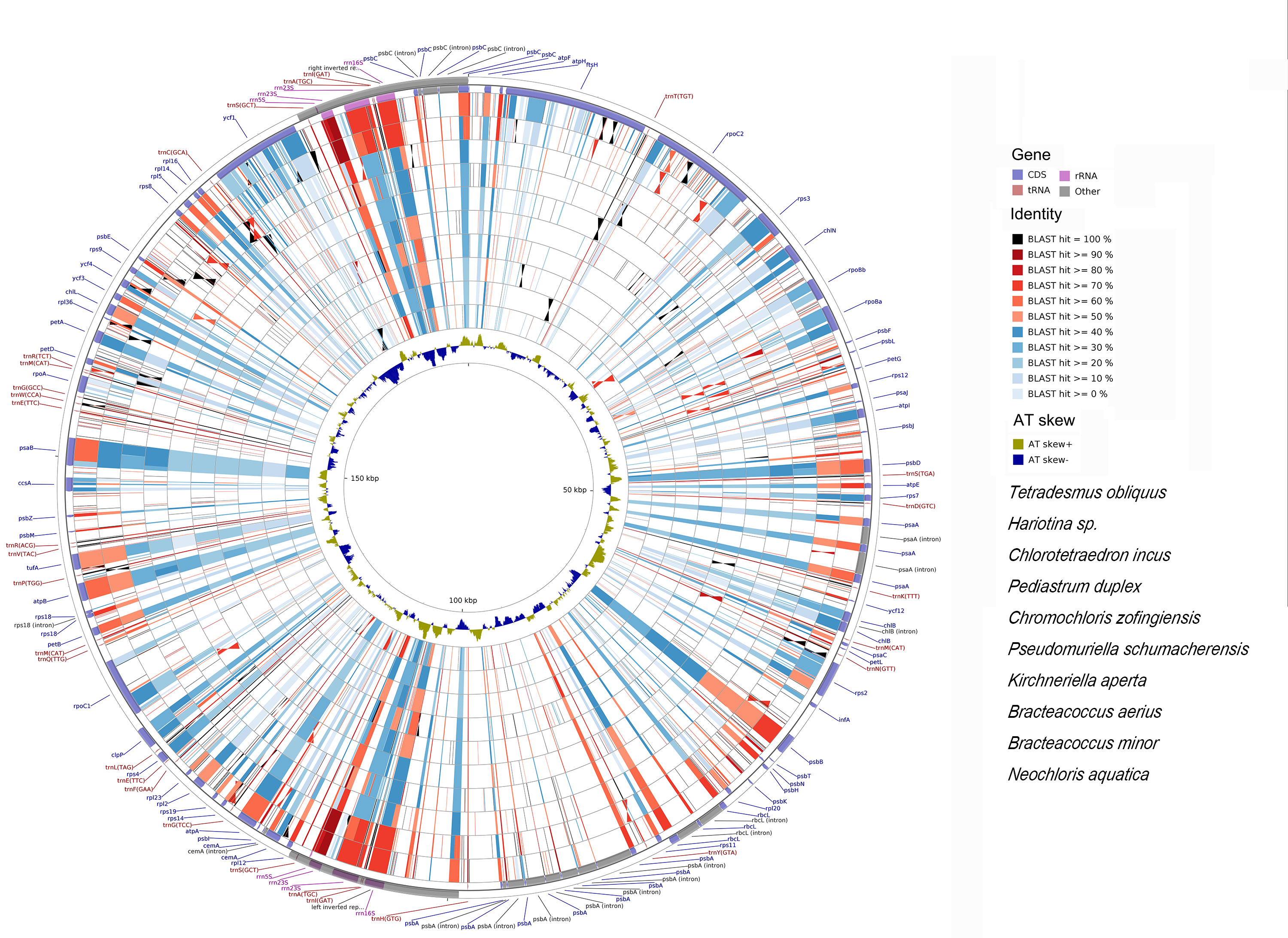

Supplement: Supplementary file 1 [file life-12-01912-s001.zip › Figure S3.tif]

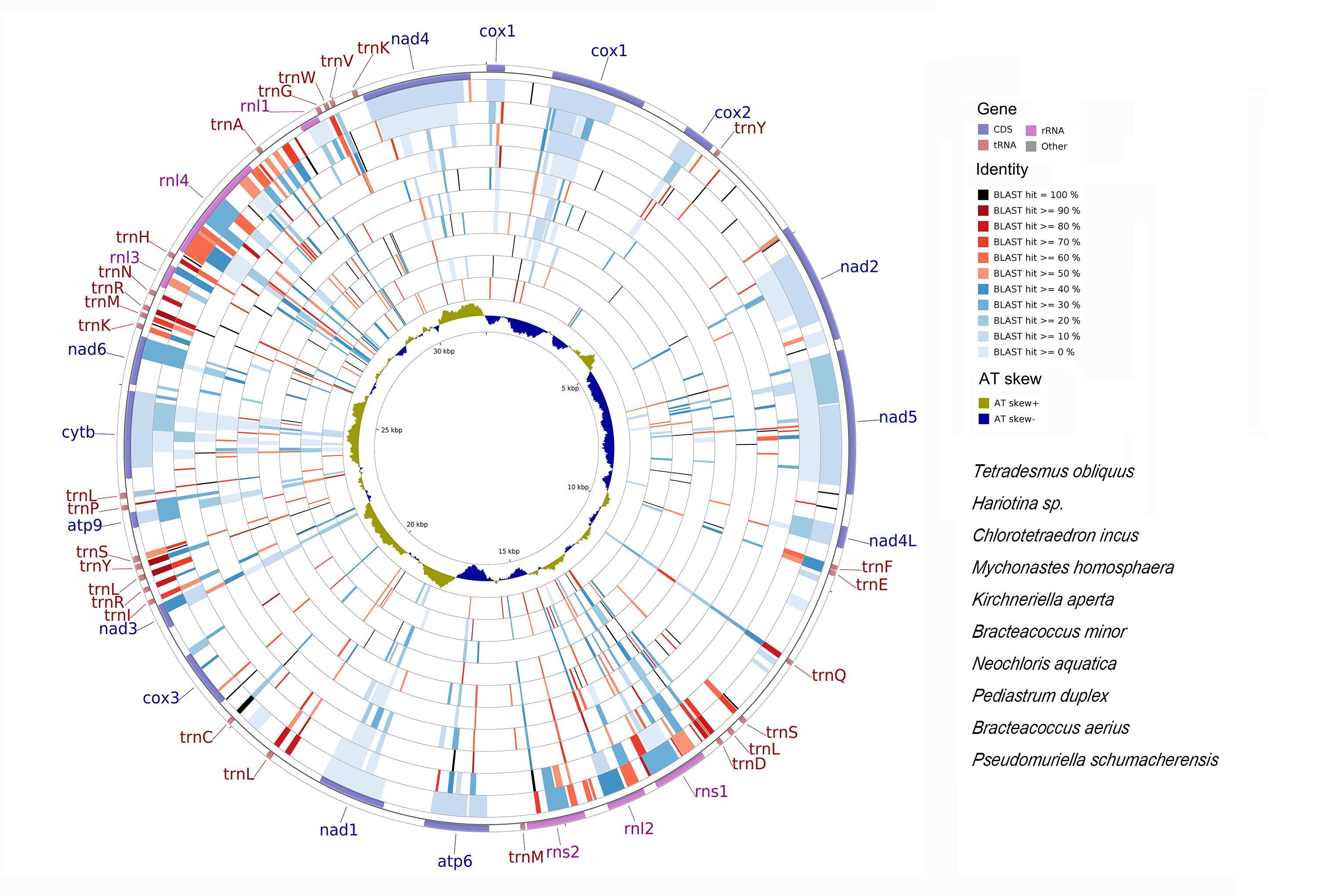

Supplement: Supplementary file 1 [file life-12-01912-s001.zip › Figure S4.tif]
